# Feasibility and impact of school-based nutrition education interventions on the diets of adolescent girls in Ethiopia: a non-masked, cluster-randomised, controlled trial

**DOI:** 10.1016/S2352-4642(23)00168-2

**Published:** 2023-10

**Authors:** Sunny S Kim, Celeste Sununtnasuk, Hanna Y Berhane, Tamirat Tafesse Walissa, Abdulaziz Ali Oumer, Yonas Taffesse Asrat, Tina Sanghvi, Edward A Frongillo, Purnima Menon

**Affiliations:** a Nutrition, Diets, and Health Unit, International Food Policy Research Institute (IFPRI), Washington, DC, USA; b Nutrition and Behavioral Sciences Department, Addis Continental Institute of Public Health, Addis Ababa, Ethiopia; c FHI Solutions, Addis Ababa, Ethiopia; d FHI Solutions, Washington, DC, USA; e Department of Health Promotion, Education, and Behavior, Arnold School of Public Health, University of South Carolina, Columbia, SC, USA; f Food and Nutrition Policy Department, IFPRI, New Delhi, India

## Abstract

**Background:**

Adolescence is a critical period of physical and psychological development, especially for girls, because poor nutrition can affect their wellbeing as well as that of their children. We aimed to assess the feasibility and impact of a package of nutrition education interventions delivered through public primary schools on the diets of adolescent girls in Ethiopia.

**Methods:**

In this non-masked, cluster-randomised, controlled trial, primary schools (clusters) in the Southern Nations, Nationalities, and People's Region and Somali region of Ethiopia were randomly allocated to the intervention group (nutrition information provided during flag ceremonies, classroom lessons, school club meetings, peer group mentoring, BMI measurement and counselling, and parent–teacher meetings) or the control group (standard academic curriculum on health and nutrition) by use of computer-generated pseudo-random numbers. Duration of the school-based interventions was 4 months, and the key messages were related to dietary diversity (eating a variety of foods), energy adequacy (eating breakfast and healthy snacks), and healthy food choices (avoiding junk foods). Adolescent girls were eligible for participation if aged 10–14 years and enrolled in grades 4–8 in a study school. Data were collected with two independent cross-sectional surveys: baseline before the start of implementation and endline 1·5 years later. The primary outcome of impact was dietary diversity score, defined as the number of food groups (out of ten) consumed over the previous 24 h using a list-based method, and minimum dietary diversity, defined as the proportion of girls who consumed foods from at least five of the ten food groups, in the intention-to-treat population. We also assessed intervention exposure as a measure of feasibility. We estimated intervention effects using linear regression models for mean differences at endline, with SEs clustered at the school level, and controlled for adolescent age, region, household food security, and wealth. The trial is registered with ClinicalTrials.Gov, NCT04121559, and is complete.

**Findings:**

27 primary schools were randomly allocated to the intervention group and 27 to the control group. Between March 22 and April 29, 2021, 536 adolescent girls participated in the endline survey (270 in the intervention group and 266 in the control group), with median age of 13·3 years (IQR 12·1–14·0). At endline, the dietary diversity score was 5·37 (SD 1·66) food groups in the intervention group and 3·98 (1·43) food groups in the control group (adjusted mean difference 1·33, 95% CI 0·90–1·75, p<0·0001). Increased minimum dietary diversity was also associated with the intervention (182 [67%] of 270 in the intervention group *vs* 76 [29%] of 266 in the control group; adjusted odds ratio 5·37 [95% CI 3·04–9·50], p<0·0001). 256 (95%) of 270 adolescent girls in the intervention group were exposed to at least one of the five in-school intervention components.

**Interpretation:**

Integrating nutrition interventions into primary schools in Ethiopia was feasible and increased dietary diversity incrementally among adolescent girls, but could be limited in changing other food choice behaviours, such as junk food consumption, based on nutrition education alone.

**Funding:**

Bill & Melinda Gates Foundation.

## Introduction

Adolescence is a crucial period of physical and psychological development and for achieving human potential. Rapid physical, psychosocial, and cognitive growth and development are coupled with increased energy and nutrient requirements.^1^, ^2^ Poor nutrition during adolescence can have adverse consequences that affect health in adulthood. Nutrition during adolescence is especially important for girls because poor nutrition can affect their wellbeing as well as the survival, health, and wellbeing of their children.^1^ Data and evidence on the nutritional status of adolescents in low-income and middle-income countries (LMICs) are scarce, even though this population includes about 1·8 billion individuals. Globally, the health and nutrition status of adolescents have improved only modestly over the past 50 years.^3^

**Research in context**

**Evidence before this study**

Adolescent nutrition has been largely overlooked in intervention and policy research. We searched PubMed from database inception to Aug 31, 2022, using the search terms (in various combinations) “adolescent”, “nutrition”, “intervention”, “diet”, “school”, “school-based”, and “education”, for intervention studies with adolescent diet or nutrition outcomes. We had filters for English language and human studies, and cross-referenced intervention studies cited in the 2022 *Lancet* Series on adolescent nutrition. We found that few randomised controlled trials and controlled before-and-after studies of school-based nutrition interventions have been conducted in low-income and middle-income countries (LMICs). The existing evidence has largely been limited to micronutrient supplementation and fortification, with few studies assessing the impact of nutrition education or counselling and macronutrient supplementation on health and nutrition outcomes among adolescents. Nutrition interventions within the education sector—arguably the best researched and most used platform—have focused on the provision of school meals, but evaluations and reviews suggest that school meals without nutritious foods and nutrition education based on behavioural science principles have minimal effect on dietary change and nutrition outcomes. In sub-Saharan Africa, evaluations of school-based nutrition interventions indicate that, although nutrition education can improve nutrition knowledge, it does not necessarily translate into healthy nutrition behaviour. Important gaps in evidence in the adolescent nutrition literature include data and research on nutrition challenges and determinants disaggregated by age, and effects of interventions that are multifaceted and address multiple challenges.

**Added value of this study**

This study evaluates a package of school-based nutrition education interventions coupled with community and health platform-based interventions and capacity building of school staff and service providers to improve the diet of adolescent girls in Ethiopia. Our sample includes adolescent girls aged 10–14 years, an age group for which data and research on nutrition challenges and determinants are scarce, but that represents a key phase of development and habit formation. Our study adds to the limited literature on the effects of school-based nutrition interventions on adolescent nutrition in LMICs. The results showed that developing a package of adolescent nutrition interventions delivered primarily through school-based platforms was feasible and improved adolescent girls’ dietary diversity and meal frequency, even with a short implementation duration (about 4 months). Junk food consumption, however, remained largely unchanged. Despite an intervention effect on the consumption of other sweets, more than half of adolescent girls reported consuming junk foods in the past 24 h.

**Implications of all the available evidence**

The findings of our study, together with those of previous studies, reflect the importance of nutrition education and the enabling environment for influencing nutrition behaviours among adolescents. Reinforcing messages about eating diverse foods and eating more often resulted in an incremental behaviour change related to dietary diversity and meal frequency; however, advising adolescents not to eat junk foods without changing their food environments appeared to be largely unsuccessful in curbing consumption of junk food. These findings suggest that nutrition knowledge is likely to have little effect without an enabling environment.

In Ethiopia, where 39% of the population is younger than 15 years,^4^ adolescent nutrition is an important public health concern. The pooled prevalence of adolescent stunting is estimated to be 21% and that of underweight (ie, low BMI for age) is estimated to be 28%, with increased risk among adolescents in rural and food insecure households.^5^ In southern Ethiopia, the prevalence of stunting among adolescent girls is more than 27%,^6^, ^7^ and a study conducted in the Damot Gale district found the prevalence of inadequate intake of nutrients—a primary determinant of undernutrition—to be greater than 80% for six key nutrients, including calcium and folate.^8^ Stunting and thinness among girls are also high in northern,^9^ northwest,^10^, ^11^ and northeastern Ethiopia.^12^ Anaemia affects 20% of adolescents aged 15–19 years nationwide, and its prevalence is likely to be higher in pastoralist regions, such as Afar and Somali, where anaemia among women is highest.^13^

Intervention studies on adolescent nutrition in Ethiopia are scarce and have largely focused on supplementation of single micronutrients, such as iron or folic acid;^14^, ^15^ however, global evidence shows that multisectoral and multifaceted strategies offer the most promise.^16^ Programmatically, schools provide an important environment to support adolescent health and nutrition because adolescents spend a large proportion of their time at school. School feeding programmes are globally the most common and long-standing interventions but have shown minimal effects on dietary change or nutrition outcomes except when coupled with nutrient-dense foods and nutrition education.^15^ Holistic school-based nutrition programmes that incorporate students and teachers as well as parents and the community have been effective in increasing consumption of nutritious foods and snacks such as fruits and vegetables, while reducing consumption of sugar-sweetened beverages and frequency of skipping breakfast.^17^ In sub-Saharan Africa, school-based interventions can have a positive effect on nutrition knowledge and micronutrient status of students,^18^ but little evidence exists for adolescent health and nutrition interventions.^19^, ^20^ In Ethiopia, two school-based adolescent nutrition education interventions suggest that peer-led behaviour change communication can improve dietary diversity among girls^21^ and that nutrition lessons, group discussions, and recipe demonstrations focused on the consumption of pulses among adolescent girls can improve knowledge and intake.^22^

Alive & Thrive is a global initiative that supports the scaling up of nutrition interventions to save lives, prevent illnesses, and contribute to healthy growth and development. In Ethiopia, Alive & Thrive has been supporting the government to achieve targets set out in its National Nutrition Program II (2016–20), and, in 2019, Alive & Thrive developed a package of locally tailored adolescent nutrition interventions to be delivered in school, household, and community settings. The core interventions involved nutrition education targeted to adolescent girls through school-based activities such as classroom lessons, flag ceremonies, school clubs, peer mentoring sessions, and BMI measurement and counselling sessions, and to their parents through parent–teacher meetings and take-home messages. The school-based interventions were delivered by existing cadres of school principals and teachers, who received training about adolescent nutrition and how to implement classroom dialogue and hands-on activities to engage adolescents and parents. Secondarily, health extension workers and influential community actors were encouraged to deliver information about adolescent nutrition to families and the broader community during health facility and home visits for other health services and at community gatherings.

We present the results of the core package of school-based nutrition education interventions delivered through public primary schools in two regions (Southern Nations, Nationalities, and People's Region [SNNPR] and Somali) in Ethiopia in terms of feasibility and impact on dietary diversity, meal frequency, and consumption of unhealthy (junk) foods in adolescent girls.

## Methods

### Study design and participants

This non-masked, cluster-randomised, controlled trial took place in two regions: SNNPR, a primarily agrarian region, and Somali, a region with a high population of pastoralists, which were selected with the Federal Government of Ethiopia and regional health bureaus. Alive & Thrive selected seven woredas (districts; four in SNNPR with 31 schools and three in the Somali region with 23 schools) as potential intervention areas on the basis of having adequate security and primary school access and infrastructure.

The study included primary schools with grades 1–8 within the government education system. A baseline survey, covering a small subsample of adolescent girls across all study schools, was conducted to examine balance between study groups and to inform intervention design and context. A smaller sample size was included at baseline due to budgetary constraints, and the study impact was intended to be estimated at endline only.

Adolescent girls were eligible for inclusion in the surveys if they were enrolled in a study primary school, were aged 10–14 years of age and enrolled in grades 4–8, and had received parental consent and given assent. Either parent of the adolescent girl (mother or father present at the time of interview) was invited to participate in the survey, and informed consent was sought from the parent before the baseline and endline surveys.

Ethical approval for this study was granted by the Institutional Review Boards of the International Food Policy Research Institute (IFPRI) in Washington, DC, USA, and the Addis Continental Institute of Public Health (ACIPH) in Addis Ababa, Ethiopia. The study protocol and the statistical analysis plan are available online.

### Randomisation and masking

The samples across the two regions were pooled to estimate intervention effects. We used stratified randomisation by woreda, to balance potential co-interventions and confounders, and by school size. Then, schools were randomly allocated to the intervention or the control group by use of computer-generated pseudo-random numbers before the baseline survey.

The evaluation study was non-masked for all participants, programme implementers, and the research team, including analysts. Adolescents and parents in the intervention group were not informed about the results of the randomisation, but there was no masking of the interventions at schools or other points of contact. At the time of enrolling and interviewing participants, assessors were not informed about intervention allocation, and the intervention allocation variable was added into the datasets at the time of data analysis after data cleaning.

### Procedures

The baseline and endline surveys included independently selected study samples. Adolescent girls were selected using a school-based sampling strategy. Lists of eligible girls were taken from primary school enrolment registries. Enrolment records (available at the time of the surveys) were used to verify the birth date or age of eligible students, which were recorded from birth certificates or parent reported. Then, adolescent girls were randomly selected by simple random sampling until the required sample size was reached, and they were visited at home to be recruited and interviewed, along with their parents. Data were collected using structured questionnaires, administered through computer-assisted personal interviewing (CAPI) by trained enumerators; CAPI forms were programmed and data were collected via SurveyCTO. At baseline and endline, school food environments were assessed via direct observation (appendix p 6). Adolescent girls were interviewed separately from their parents to provide privacy and reduce cues for response bias.

In February, 2020, following the baseline survey, Alive & Thrive conducted training for principals, teachers, and health extension workers participating in the intervention. Intervention materials (instruction manuals; adolescent nutrition passports; BMI measurement guides; and posters on eating breakfast, healthy snacks, and dietary diversity) were also distributed. However, in March, 2020, the first COVID-19 case was reported in Ethiopia, and the country declared a state of emergency and closed all schools for the remainder of the 2019–20 academic year. All programme activities were halted, and interventions were not delivered to students and parents. Schools reopened for the 2020–21 academic year, and classes resumed in the study schools by October. In November, 2021, Alive & Thrive reinitiated the programme with refresher training for all principals, teachers, and health extension workers in the intervention group. The duration of the school-based interventions was one school semester (about 4 months).

Alive & Thrive's core package of school-based interventions included six components: weekly flag ceremonies, weekly classroom lessons, weekly school club meetings, weekly peer group mentoring, BMI measurement and counselling (once per semester), and monthly parent–teacher meetings (appendix p 7). An action point included in all intervention components targeted to adolescent girls was to share what they heard or learned with their parents, so that parents’ meetings were not the only source of nutrition information for parents. Each intervention component included key nutrition messages related to dietary diversity (eating a variety of foods), energy adequacy (eating breakfast and healthy snacks), and healthy food choices (avoiding unhealthy or so-called junk foods). Intervention materials were designed to reflect locally appropriate language and images. Given that promoting all the diverse food groups might have been be too much information, interventions reinforced some locally available food examples that were less frequently consumed, such as roasted mix of beans and nuts, banana, mango, milk, egg, and meat or fish.

In addition to the school-based interventions, health extension workers (working in the same area as an intervention school) were instructed to provide the key intervention messages about adolescent nutrition during routine home visits for other health services at households in which adolescent girls lived. Health extension workers and influential community actors such as community and religious leaders also provided adolescent nutrition messages during community gatherings. These secondary interventions were not considered core interventions of the programme, were conducted irregularly, and reached minimal exposure, and were, therefore, excluded from the results in this paper.

Participants in the control group received standard academic curriculum on health and nutrition, provided as part of the government education system. School principals and teachers were not instructed, supervised, or followed up about adolescent nutrition activities. No additional guidance or support was provided to the control schools.

### Outcomes

The primary outcome of impact was dietary diversity, defined as the number of food groups out of ten (grains, white roots, and tubers; pulses such as beans, peas, and lentils; nuts and seeds; dairy; meat, poultry, and fish; eggs; dark green leafy vegetables; other vitamin A-rich fruits and vegetables; other vegetables; and other fruits) consumed by adolescent girls over the previous 24 h, and minimum dietary diversity.^23^ Adolescent girls were asked about consumption of each of the ten food groups during the day and night in the previous 24 h using a list-based method, which included local food examples from the Diet Quality Questionnaire adapted for Ethiopia.^24^ Minimum dietary diversity, or the proportion of girls who consumed foods from at least five of the ten food groups, was also calculated as part of the primary outcome to assess diet quality.^23^ Although minimum dietary diversity is a proxy indicator for higher micronutrient adequacy for women aged 15–49 years, we have applied this indicator for our study sample of girls aged 10–14 years in the absence of a specific indicator for this age group.

Secondary outcomes were meal frequency and consumption of junk foods. Meal frequency (score of 0–6) was calculated as the number of times meals and snacks were consumed during the day and night in the previous 24 h both before the last school day and the last non-school day. Adolescent girls were asked whether they consumed any food during six meal and snack times: breakfast, snacks after breakfast but before a mid-day meal, lunch, afternoon snacks, dinner, and after-dinner snacks. Junk food consumption included four categories: baked sweets, other sweets (eg, candy and chocolate), fried and salty foods, and soda and sugar-sweetened beverages.^24^ Adolescent girls were asked whether they consumed food examples from each of these four categories in the previous 24 h; junk food consumption was defined as consumption of any one of these categories. Indicators of nutrition knowledge were estimated as the percentage of adolescents and parents who could name at least five different food groups (dietary diversity), knew the recommended number of meals and snacks per day (meal frequency), and identified avoiding junk foods as part of good nutrition practice (junk food consumption).

To assess feasibility, exposure to each of the six intervention components (flag ceremonies, classroom lessons, school club meetings, peer group mentoring, BMI measurement and counselling, and parent–teacher meetings) was calculated as the proportion of adolescent girls or parents who reported receiving or participating in the interventions in the past 3 months (secondary outcome). We identified barriers and opportunities related to intervention implementation based on the exposure results.

The original protocol was amended (on March 9, 2021) to remove a quantitative 24 h recall dietary assessment, the corresponding outcome indicators on adequate intake of micronutrients and macronutrients, and a sub-sample panel of adolescent girls at endline due to budgetary and time constraints.

No adverse events were anticipated or monitored actively, and none were reported by teachers or other school staff during routine supervision of intervention activities.

### Statistical analysis

The sample size was estimated based on a mean dietary diversity score of 3·26 (SD 1·40) using the women's dataset from the Alive & Thrive phase 1 endline survey for SNNPR, assuming an intra-cluster correlation (ICC) of 0·18, total cluster number of 54, 80% power to detect differences, and an α value of 0·05. We calculated that a sample size of 189 girls per group (seven per cluster) would be able to detect a minimum difference of 0·6 food groups. The sample size was rounded to 270 girls per group so that ten girls per school (two per grade) would be sampled, for a total sample size of 540 adolescent girls.

Based on our baseline data (small subsample), we observed a mean dietary diversity score of 3·69 (SD 1·56) and an ICC of 0·015; using this information, we estimated that this sample size for the endline would detect a minimum difference of 0·5 food groups with 93% power.

Descriptive analyses were conducted for sample characteristics and exposure to intervention components. Control variables used in the analyses included household wealth and household food security. Data on household asset ownership and housing characteristics were used to construct a wealth index using principal component analysis.^25^ Study households were then separated into wealth terciles based on their wealth rank. The Household Food Insecurity Access Scale^26^ was used to measure the access component of household food security and define households as food secure, mildly food insecure, moderately food insecure, or severely food insecure.

The primary analysis was performed in the intention-to-treat population at endline. We estimated intervention effects using linear regression models for mean differences, with SEs clustered at the school level obtained using a sandwich estimator (unadjusted model). For binary outcomes, we reported intervention effects as odds ratios (ORs) and 95% CIs estimated from logistic regression models. In adjusted models, we controlled for adolescent age, region, household food security, and wealth. We examined a dose–response effect in the intervention group only by using multivariable regression analyses to test associations between exposure to the number of intervention components or the individual components and the primary and secondary outcomes, adjusted for adolescent age and geographical clustering. Two-sided *t* tests were used to compare and infer significant differences between study groups at endline. For categorical variables, Pearson's χ^2^ tests were performed. All statistical analysis was done using Stata (version 17.0).

This trial is registered at ClinicalTrials.Gov, NCT04121559, and is complete.

### Role of the funding source

The funder of the study had no role in study design, data collection, data analysis, data interpretation, or writing of the report.

## Results

27 primary schools were randomly allocated to the intervention group (with a median of 625 [IQR 424–1004] pupils) and 27 to the control group (with a median of 641 [375–1141] pupils). 162 in-school adolescent girls participated in the baseline survey between Oct 8 and Nov 4, 2019, and 536 adolescent girls (median age 13·3 years [IQR 12·1–14·0]) participated in the endline survey between March 22 and April 29, 2021 (figure). All 54 primary schools were included at baseline and endline, with no clusters lost to follow-up. At baseline, demographic characteristics were similar between study groups (table 1). At endline, 518 (97%) of the 536 adolescent girls surveyed were students returning to the same schools, so very few were newly enrolled in schools at endline, and households in the study areas were likely to have remained in place over the previous year.

**Figure:**
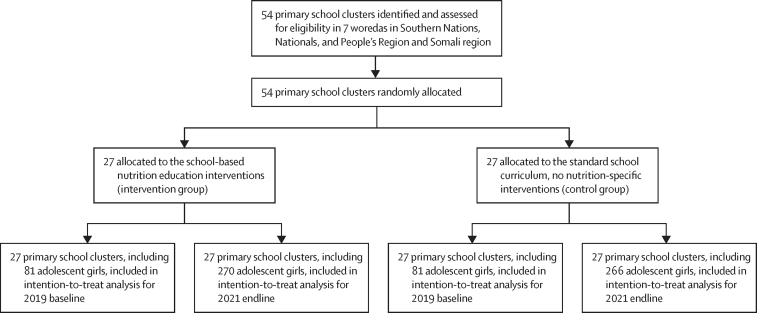
Trial profile

**Table 1:** Sample characteristics

			**2019 baseline**		**2021 endline**		
			Intervention group	Control group	Intervention group	Control group	p value*
Primary schools			27	27	27	27	..
	Primary school characteristics						
		Number with a toilet facility available	24 (89%)	24 (89%)	27 (100%)	25 (93%)	0·16
		Number of classrooms per school	12·0 (6·2)	11·3 (5·5)	14·0 (9·8)	12·4 (5·5)	0·48
		Number of teachers per school	18·4 (13·1)	16·3 (10·0)	19·2 (13·4)	17·0 (10·6)	0·49
Adolescent girls surveyed			81	81	270	266	..
	Adolescent dietary diversity						
		Dietary diversity score (0–10 food groups)	3·80 (1·59)	3·58 (1·52)	5·37 (1·66)	3·98 (1·43)	<0·0001
		Minimum dietary diversity (≥5 food groups)	24 (30%)	19 (23%)	182 (67%)	76 (29%)	<0·0001
	Meal frequency in past 24 h (0–6)		..	..	4·0 (0·9)	3·2 (0·9)	<0·0001
	Junk food consumption in past 24 h		50 (62%)	48 (59%)	131 (49%)	146 (55%)	0·29
	Age, years		..	..	..	..	0·27
		Mean (SD)	12·6 (1·2)	12·8 (1·2)	12·9 (1·2)	13·0 (1·2)	..
		Median (IQR)	12·9 (12·0–13·4)	13·1 (12·0–13·9)	13·3 (12·1–14·0)	13·4 (12·3–14·0)	..
	BMI, kg/m^2^		17·2 (2·5)	17·4 (2·2)	17·4 (2·7)	17·3 (2·5)	0·84
	Grade level		..	..	..	..	0·99
		4	8 (10%)	12 (15%)	53 (20%)	57 (21%)	..
		5	15 (19%)	20 (25%)	55 (20%)	53 (20%)	..
		6	19 (23%)	18 (22%)	56 (21%)	55 (21%)	..
		7	18 (22%)	22 (27%)	53 (20%)	51 (19%)	..
		8	21 (26%)	9 (11%)	53 (20%)	50 (19%)	..
	Languages spoken						
		Amharic	47 (58%)	38 (47%)	156 (58%)	128 (48%)	0·45
		Gurage	25 (31%)	22 (27%)	81 (30%)	76 (29%)	0·90
		Somali	32 (40%)	36 (44%)	109 (40%)	116 (44%)	0·81
		Other	22 (27%)	22 (27%)	77 (29%)	72 (27%)	0·90
	Residence of parents						
		Father currently resides with adolescent	63 (78%)	67 (83%)	201 (74%)	218 (82%)	0·046
		Mother currently resides with adolescent	78 (96%)	79 (98%)	251 (93%)	252 (95%)	0·47
Parents surveyed			81	81	270	266	..
	Parent respondent type		..	..	..	..	0·84
		Father	16 (20%)	10 (12%)	65 (24%)	66 (25%)	..
		Mother	65 (80%)	71 (88%)	205 (76%)	200 (75%)	..
	Father's age, years		44·8 (13·8)	46·7 (11·6)	48·9 (10·7)	48·8 (11·3)	0·98
	Father's education level†		..	..	..	..	0·72
		Never attended school	3 (19%)	1 (10%)	18 (28%)	21 (32%)	..
		Primary school (grades 1–8)	11 (69%)	5 (50%)	35 (54%)	36 (55%)	..
		Secondary school or higher	2 (13%)	4 (40%)	12 (18%)	9 (14%)	..
	Father's occupation		..	..	..	..	0·0075
		Farmer	9 (56%)	7 (70%)	46 (71%)	59 (89%)	..
		Other	7 (44%)	3 (30%)	19 (29%)	7 (11%)	..
	Mother's age, years		38·3 (9·4)	39·7 (8·1)	38·6 (7·9)	39·8 (7·9)	0·13
	Mother's education level†		..	..	..	..	0·045
		Never attended school	31 (48%)	38 (54%)	101 (49%)	115 (58%)	..
		Primary school (grades 1–8)	29 (45%)	33 (46%)	81 (40%)	75 (38%)	..
		Secondary school or higher	5 (8%)	0 (0%)	23 (11%)	10 (5%)	..
	Mother's occupation		..	..	..	..	0·12
		Housewife	33 (51%)	37 (52%)	94 (46%)	96 (48%)	..
		Farmer	12 (18%)	18 (25%)	46 (23%)	58 (29%)	..
		Other	20 (31%)	16 (23%)	64 (31%)	46 (23%)	..
	Household Food Insecurity Access Scale		..	..	..	..	0·016
		Food secure	35 (43%)	36 (44%)	159 (59%)	132 (50%)	..
		Mildly insecure	7 (9%)	8 (10%)	23 (9%)	15 (6%)	..
		Moderately insecure	27 (33%)	29 (36%)	60 (22%)	71 (27%)	..
		Severely insecure	12 (15%)	8 (10%)	28 (10%)	48 (18%)	..
	Wealth tercile		..	..	..	..	0·0048
		Low	29 (36%)	25 (31%)	85 (31%)	94 (35%)	..
		Middle	25 (31%)	29 (36%)	78 (29%)	101 (38%)	..
		High	27 (33%)	27 (33%)	107 (40%)	71 (27%)	..

Data are n, mean (SD), or median (IQR) unless otherwise stated.

* Differences between study groups at endline, accounting for clustering, were calculated using two-sided *t* tests. For categorical variables, Pearson's χ^2^ tests were performed.

† At endline, parents (two fathers and four mothers) who attended religious school are included in the “Never attended school” category.

For the primary outcome of dietary diversity, we observed an adjusted mean difference of 1·33 (95% CI 0·90–1·75, p<0·0001) food groups in the dietary diversity score between the intervention (mean 5·37 [SD 1·66]) and control (3·98 [1·43]) groups at endline (table 2). The intervention was also associated with an increase in minimum dietary diversity (consuming at least five food groups; 182 [67%] of 270 in the intervention group *vs* 76 [29%] of 266 in the control group; adjusted OR 5·37 [95% CI 3·04–9·50], p<0·0001). A breakdown of the individual food groups consumed reflects an intervention effect on seven of the ten food groups, including those foods promoted by the interventions.

**Table 2:** Dietary diversity, consumption of junk foods, and meal frequency among adolescent girls at endline

		**Endline**		**Unadjusted**		Adjusted*	
		Intervention group (n=270)	Control group (n=266)	β or odds ratio (95% CI)	p value	β or odds ratio (95% CI)	p value
Dietary diversity							
	Dietary diversity score† (0–10 food groups)	5·37 (1·66)	3·98 (1·43)	1·39 (0·90–1·88)	<0·0001	1·33 (0·90–1·75)	<0·0001
	Minimum dietary diversity† (≥5 food groups)	182 (67%)	76 (29%)	5·17 (2·94–9·10)	<0·0001	5·37 (3·04–9·50)	<0·0001
Food groups consumed in the past 24 h							
	Grains, white roots, and tubers	264 (98%)	264 (99%)	0·33 (0·07–1·55)	0·16	0·25 (0·06–1·05)	0·058
	Pulses (beans, peas, and lentils)	199 (74%)	148 (56%)	2·23 (1·39–3·60)	0·0010	2·39 (1·51–3·76)	0·0002
	Nuts and seeds	68 (25%)	35 (13%)	2·22 (1·02–4·84)	0·044	3·10 (1·52–6·32)	0·0018
	Dairy	146 (54%)	97 (36%)	2·05 (0·95–4·41)	0·066	3·09 (1·53–6·27)	0·0017
	Meat, poultry, and fish	67 (25%)	31 (12%)	2·50 (1·16–5·40)	0·019	3·42 (2·02–5·80)	<0·0001
	Eggs	113 (42%)	45 (17%)	3·53 (1·79–6·98)	0·0003	4·14 (2·06–8·30)	<0·0001
	Dark green leafy vegetables	130 (48%)	121 (45%)	1·11 (0·55–2·26)	0·77	1·03 (0·56–1·89)	0·94
	Other vitamin A-rich fruits and vegetables	99 (37%)	37 (14%)	3·58 (2·04–6·30)	<0·0001	3·44 (1·97–6·02)	<0·0001
	Other vegetables	252 (93%)	237 (89%)	1·71 (0·81–3·61)	0·16	1·53 (0·75–3·11)	0·24
	Other fruits	112 (41%)	44 (17%)	3·58 (1·93–6·62)	<0·0001	3·32 (1·84–6·00)	<0·0001
Meal frequency in past 24 h (0–6)†		4·04 (0·92)	3·17 (0·88)	0·87 (0·61–1·13)	<0·0001	0·84 (0·58–1·09)	<0·0001
Meal or snack times, %							
	Breakfast	238 (88%)	199 (75%)	2·50 (1·12–5·57)	0·025	2·32 (1·05–5·15)	0·038
	Morning snack	141 (52%)	44 (17%)	5·51 (2·85–10·69)	<0·0001	6·45 (3·57–11·66)	<0·0001
	Lunch	253 (94%)	242 (91%)	1·48 (0·61–3·54)	0·38	1·30 (0·56–2·98)	0·54
	Afternoon snack	168 (62%)	84 (32%)	3·57 (2·23–5·72)	<0·0001	3·60 (2·26–5·75)	<0·0001
	Dinner	262 (97%)	259 (97%)	0·89 (0·31–2·52)	0·82	0·70 (0·23–2·10)	0·52
	Evening snack	28 (10%)	15 (6%)	1·94 (0·88–4·26)	0·10	2·07 (0·93–4·58)	0·074
Consumed any junk foods in past 24 h†		131 (49%)	146 (55%)	0·77 (0·49–1·23)	0·28	0·73 (0·46–1·14)	0·17
Junk foods consumed in the past 24 h							
	Baked sweets	36 (13%)	38 (14%)	0·92 (0·45–1·88)	0·83	0·91 (0·47–1·79)	0·79
	Other sweets (candy or chocolate)	38 (14%)	61 (23%)	0·55 (0·32–0·95)	0·03	0·49 (0·29–0·83)	0·0085
	Fried and salty foods	29 (11%)	37 (14%)	0·74 (0·42–1·33)	0·32	0·72 (0·41–1·28)	0·27
	Soda and sugar-sweetened beverages	88 (33%)	92 (35%)	0·91 (0·57–1·47)	0·71	0·92 (0·61–1·40)	0·70

Data are mean (SD) or n (%) unless otherwise stated. Differences between study groups are by intention-to-treat analyses, accounting for clustering using linear regression models for mean differences, with SEs clustered at the school level obtained using a sandwich estimator. For binary outcomes, intervention effects are reported as odds ratios and 95% CIs estimated from logistic regression models.

* Linear or logistic regression, adjusted for adolescent age, region, household food security, and wealth, clustered by school.

† Intra-cluster correlation coefficients: dietary diversity score (0·392), minimum dietary diversity (0·284), meal frequency in past 24 h (0·350), and consumed any junk foods in past 24 h (0·102).

For meal frequency in the past 24 h, we observed an adjusted mean difference of 0·84 (95% CI 0·58–1·09, p<0·0001) meal or snack times between study groups (4·04 [SD 0·92] in the intervention group *vs* 3·17 [0·88] in the control group) at endline (table 2). Improvements were observed in the breakfast and morning and mid-afternoon snacks times. There was no significant difference in consumption of junk foods in the past 24 h between study groups (131 [49%] of 270 in the intervention group *vs* 146 [55%] of 266 in the control group; adjusted OR 0·73, 95% CI 0·46–1·14, p=0·17). A breakdown of the specific categories of unhealthy foods showed an intervention effect on the consumption of other sweets such as candies and chocolates only (38 [14%] of 270 girls in the intervention group *vs* 61 [23%] of 266 girls; difference 8·8 percentage points; adjusted OR 0·49, 95% CI 0·29–0·83, p=0·0085).

Both adolescent girls and their parents in the intervention group had higher knowledge at endline than those in the control group about dietary diversity, meal frequency, and consumption of unhealthy foods (table 3). Adolescent girls had more knowledge about meal frequency than about dietary diversity or avoiding unhealthy foods; similar patterns were observed among their parents.

**Table 3:** Prevalence of correct knowledge about nutrition among adolescent girls and parents at endline

	**Endline**		**Unadjusted**		Adjusted*	
	Intervention group (n=270)	Control group (n=266)	Odds ratio (95% CI)	p value	Odds ratio (95% CI)	p value
**Adolescent girls**						
Knows ≥5 food groups of a diverse diet	111 (41%)	26 (10%)	6·44 (3·56–11·66)	<0·0001	6·43 (3·54–11·68)	<0·0001
Knows to eat ≥4 times per day, ≥3 meals per day, and ≥1 snack per day	230 (85%)	120 (45%)	7·00 (3·73–13·13)	<0·0001	6·80 (3·70–12·50)	<0·0001
Knows avoiding junk food and sugar-sweetened beverages is good nutrition	118 (44%)	36 (14%)	4·96 (2·79–8·80)	<0·0001	4·93 (2·74–8·86)	<0·0001
**Parents**						
Knows ≥5 food groups of a diverse diet	95 (35%)	35 (13%)	3·58 (2·16–5·95)	<0·0001	3·70 (2·21–6·17)	<0·0001
Knows to eat ≥4 times per day, ≥3 meals per day, and ≥1 snack per day	218 (81%)	120 (45%)	5·10 (2·82–9·21)	<0·0001	4·71 (2·63–8·46)	<0·0001
Knows avoiding junk food and sugar-sweetened beverages is good nutrition	89 (33%)	40 (15%)	2·78 (1·71–4·51)	<0·0001	2·84 (1·69–4·75)	<0·0001

Data are n (%) unless otherwise stated. Differences between study groups in intention-to-treat analyses, accounting for clustering using logistic regression models.

* Logistic regression, adjusted for adolescent age, region, household food security, and wealth, clustered by school.

At endline, free school meals were not provided, nearly all study schools had at least one food vendor within 1 min walking distance (52 of 54 schools), and about a quarter of all adolescent girls had seen or heard a food advertisement in the past 3 months (appendix p 6). The school-based intervention was highly successful in reaching adolescent girls and their parents. 256 (95%) of 270 adolescent girls in the intervention group were exposed to at least one of the five in-school intervention components (table 4). Adolescent girls in the intervention group most commonly received nutrition information via flag ceremonies (230 [85%]), classroom lessons (226 [84%]), and BMI measurements (197 [73%]). 145 (54%) of 270 parents of adolescent girls in the intervention group were also exposed to discussions about nutrition at parents’ meetings at schools.

**Table 4:** Exposure to interventions in the past 3 months among adolescent girls and their parents at endline

		**Endline**		**Unadjusted**		Adjusted*	
		Intervention group (n=270)	Control group (n=266)	Odds ratio (95% CI)	p value	Odds ratio (95% CI)	p value
Intervention							
	Heard about nutrition during flag ceremony	230 (85%)	41 (15%)	31·55 (15·98–62·32)	<0·0001	33·43 (16·93–66·00)	<0·0001
	Heard about nutrition in the classroom	226 (84%)	61 (23%)	17·26 (9·20–32·40)	<0·0001	18·66 (9·58–36·33)	<0·0001
	Participated in a club about nutrition for girls	121 (45%)	7 (3%)	30·05 (10·48–86·16)	<0·0001	30·43 (9·74–95·09)	<0·0001
	Participated in a mentorship programme for girls	120 (44%)	6 (2%)	34·67 (13·18–91·18)	<0·0001	38·20 (13·89–105·11)	<0·0001
	BMI measurements taken at school	197 (73%)	1 (<1%)	715·14 (97·24–5259·20)	<0·0001	830·46 (108·54–6353·91)	<0·0001
	Nutrition discussed at school parents' meeting	145 (54%)	12 (5%)	24·55 (13·00–46·37)	<0·0001	27·19 (13·66–54·11)	<0·0001
Exposed to ≥1 interventions		256 (95%)	85 (32%)	38·94 (11·29–134·24)	<0·0001	42·58 (12·92–140·40)	<0·0001

Data are n (%) unless otherwise stated. Differences between study groups in intention-to-treat analyses, accounting for clustering using logistic regression models.

* Logistic regression, adjusted for adolescent age, region, household food security, and wealth, clustered by school.

There was an association between exposure to multiple intervention components and improved outcomes (appendix p 8). We observed that exposure to at least two intervention components was associated with substantially higher odds of achieving minimum dietary diversity and high meal frequency among adolescent girls. Although we observed some associations between exposure to individual intervention components and improved outcomes, there was too much overlap in the exposure to individual components to draw conclusions on their comparative effects (appendix p 9).

## Discussion

In this non-masked, cluster-randomised, controlled trial, a package of school-based nutrition education interventions delivered during one school semester (about 4 months) improved practices and knowledge related to dietary diversity and meal frequency among young adolescent girls. High reported exposure to the intervention components was achieved in the intervention group. We observed lower consumption of sweets in the previous 24 h, but no impact on consumption of any other junk foods.

The diets of adolescent girls in our study had low diversity overall. Low dietary diversity among adolescent girls aged 10–19 years (broader than the 10–14 year age range in our study) was corroborated in a recent observational study in SNNPR, one of our study regions; the mean dietary diversity score was 3·6 food groups, and only 28% of participants consumed at least five food groups.^27^ Each of the Alive & Thrive intervention components was designed to address information about locally available foods that make up a diverse diet, particularly specific food groups with large room for improvement based on formative study, and provide motivation to acquire and consume a diverse diet. After the interventions, we observed an impact of about one food group difference between study groups, and minimum dietary diversity increased from 30% at baseline to 67% at endline in the intervention group. A study in Jimma zone, Oromia region, showed that peer-led behaviour change communication through school media and health clubs increased minimum dietary diversity among school adolescents aged 10–19 years from 35% at baseline to 75% at endline in the intervention group.^21^ Additionally, a 6-month school nutrition education intervention (bi-monthly lessons with recipes and tastings) that specifically focused on pulse consumption among adolescent girls in Halaba district, SNNPR, improved knowledge and intake of pulses.^22^ Both of these studies included predominantly farming and food insecure households,^21^, ^22^ similar to our study households. These studies as well as our study indicated that, if designed to address behavioural determinants such as knowledge, attitudes, and beliefs, in-school nutrition education interventions might be effective in improving diversified food or specific food consumption among adolescents, even in rural and food insecure contexts.

Meal frequency knowledge was higher among adolescent girls and their parents in both study groups compared with knowledge that at least five food groups make up a diverse diet and that avoiding junk food and sugar-sweetened beverages is part of good nutrition. However, meal frequency was three meals per day on average in the control areas at endline; this was similar to findings from an observational study in Damot Sore district, SNNPR, in which 76% of 719 adolescent girls aged 10–19 years consumed three or fewer meals per day.^7^ We observed an intervention effect of nearly one meal or snack consumed, mainly due to an increase in the consumption of breakfast and mid-morning or mid-afternoon snacks. Improvement in these meal or snack periods aligned with the key intervention messages of consuming breakfast before school and eating a healthy snack every day.

Despite an intervention effect on the consumption of other sweets, more than half of the adolescent girls reported consuming junk food, with no differences in the consumption of fried and salty foods, baked sweets, or soda and sugar-sweetened beverages between study groups. Knowledge about avoiding junk food as part of good nutrition improved among adolescent girls and their parents between study groups, but knowledge did not translate into practice. This result might not be surprising, considering that consumption of unhealthy foods is influenced by a complex set of factors. Junk foods are designed to be tasty, convenient (ready-to-eat packaging), and easily available, and are subject to commercial advertisements. At endline, nearly all study schools had at least one food vendor within 1 min walking distance, mainly selling sweets and soda or sugar-sweetened beverages, and about a quarter of adolescent girls had seen or heard a food advertisement in the past 3 months. Therefore, nutrition education and telling adolescents not to eat junk foods that they crave or enjoy without changing their food environments might not be successful in curbing junk food consumption.

Although it was not possible to disentangle the effects of individual components within our intervention package, exposure to at least two components was associated with improved outcomes. A previous study on the association between the combination of behaviour change interventions or number of contacts and child feeding practices showed that results are context specific;^28^ however, using multiple social and behaviour change communication activities and channels to change behaviours might be more effective than using one.^29^ In our study, the frequency of the six core intervention components varied from weekly to monthly, and the effect of multiple components reinforcing the key intervention messages probably contributed to the improved dietary practices.

Parents had an essential role in the diets of young adolescents in our study. Although the adolescent girls spent time away from their parents throughout the school day, parents were still the primary source of and gatekeepers to their foods. Free school meals were not provided in any of our study schools, and only one intervention school had a canteen but no food items were observed, so adolescents were limited to foods from home, foods received from peers or others, or foods purchased from around the home or school premises. Nutrition education targeted to, and active involvement of, parents are crucial components of adolescent nutrition interventions. Evidence of improved food preparation by parents for their adolescent daughters and their home food environment were observed in the intervention group at endline.

Our study addresses the important evidence gap on the effectiveness of nutrition education interventions to improve adolescents’ dietary practices, particularly in Ethiopia. Our randomised design lends confidence in attributing the observed impact and changes in outcomes to the interventions. Furthermore, the package of school-based interventions was relatively low cost to develop and implement. Total estimated programme costs (including programme staff salaries, materials and other resources, transportation, non-governmental organisation budget, and school staff salary) for programme reach per beneficiary was roughly US$43·22 per adolescent girl (grades 4–8) in the intervention schools, or, if considered school wide, $13·79 per student (boys and girls in grades 1–8).

Study data collected were based on self-reports, which are subject to recall and social desirability bias. To minimise these biases, measures for the primary and secondary outcomes used locally available food examples or specific meal or snack times throughout the day with a short 24 h recall period. Adolescent girls were interviewed separately from their parents and the variation in intervention effects across outcomes also suggests that adolescents might not be reporting socially desirable responses. No hard clinical outcomes were included in this study because the endpoints were dietary behaviours. In relation to adherence among study participants, we assessed implementation fidelity by measuring exposure to programme inputs, delivery of interventions by school staff, and exposure to each intervention component among adolescent girls and their parents over the intervention period. Moreover, the primary analysis to assess effectiveness was prespecified as intention-to-treat analysis at endline; therefore, all participants remained in the study sample for analysis, regardless of adherence to the protocol. Our study was conducted during the COVID-19 pandemic, which affected school schedules and activities; however, there were no differences in the school conditions by study group. Selection bias is possible given that the schools were randomly allocated and unmasked before participants were enrolled in the endline survey, but we consider this bias to be minimal given that interventions were not rolled out to students and parents before the pandemic and took place after school enrolment at the start of the new academic year.

In conclusion, a package of nutrition education interventions delivered in primary schools in Ethiopia was feasible to implement and effective in improving adolescent girls’ dietary practices. Reinforcing messages about eating diverse foods and eating more often resulted in incremental behaviour change related to dietary diversity and meal frequency; however, informing adolescents to avoid junk foods, without addressing their food environments, was not effective in reducing junk food consumption. Other intervention strategies might be required to change food environments, such as the provision or product placement of healthy food or snack options at or near schools, and behaviours particularly related to consumption of junk foods, which is a growing concern among adolescents even in the rural areas in Ethiopia, as in many other contexts.

## Data sharing

De-identified participant data, data dictionaries, and questionnaires from the endline survey have been submitted for open access publication and will be available on Harvard Dataverse.

## Declaration of interests

We declare no competing interests.

## References

[1] Das JK, Salam RA, Thornburg KL (2017). Nutrition in adolescents: physiology, metabolism, and nutritional needs. Ann NY Acad Sci.

[2] Spear BA (2002). Adolescent growth and development. J Am Diet Assoc.

[3] Akseer N, Al-Gashm S, Mehta S, Mokdad A, Bhutta ZA (2017). Global and regional trends in the nutritional status of young people: a critical and neglected age group. Ann NY Acad Sci.

[4] United Nations Population Fund (2022). World Population Dashboard Ethiopia. https://www.unfpa.org/data/world-population/ET.

[5] Berhe K, Kidanemariam A, Gebremariam G, Gebremariam A (2019). Prevalence and associated factors of adolescent undernutrition in Ethiopia: a systematic review and meta-analysis. BMC Nutr.

[6] Handiso YH, Belachew T, Abuye C, Workicho A, Baye K (2021). Undernutrition and its determinants among adolescent girls in low land area of Southern Ethiopia. PLoS One.

[7] Gagebo DD, Kerbo AA, Thangavel T (2020). Undernutrition and associated factors among adolescent girls in Damot Sore District, Southern Ethiopia. J Nutr Metab.

[8] Yilma B, Endris BS, Mengistu YG, Sisay BG, Gebreyesus SH (2021). Inadequacy of nutrient intake among adolescent girls in south central Ethiopia. J Nutr Sci.

[9] Berhe K, Gebremariam G (2020). Magnitude and associated factors of undernutrition (underweight and stunting) among school adolescent girls in Hawzen Woreda (District), Tigray regional state, Northern Ethiopia: cross-sectional study. BMC Res Notes.

[10] Tariku A, Belew AK, Gonete KA (2019). Stunting and its determinants among adolescent girls: findings from the Nutrition Surveillance Project, Northwest Ethiopia. Ecol Food Nutr.

[11] Kebede D, Prasad RPCJ, Asres DT, Aragaw H, Worku E (2021). Prevalence and associated factors of stunting and thinness among adolescent students in Finote Selam Town, Northwest Ethiopia. J Health Popul Nutr.

[12] Hadush G, Seid O, Wuneh AG (2021). Assessment of nutritional status and associated factors among adolescent girls in Afar, Northeastern Ethiopia: a cross-sectional study. J Health Popul Nutr.

[13] Central Statistical Agency Ethiopia (2016). Ethiopia Demographic and Health Survey 2016. https://dhsprogram.com/pubs/pdf/FR328/FR328.pdf.

[14] Salam RA, Hooda M, Das JK (2016). Interventions to improve adolescent nutrition: a systematic review and meta-analysis. J Adolesc Health.

[15] Lassi ZS, Moin A, Das JK, Salam RA, Bhutta ZA (2017). Systematic review on evidence-based adolescent nutrition interventions. Ann NY Acad Sci.

[16] Hargreaves D, Mates E, Menon P (2022). Strategies and interventions for healthy adolescent growth, nutrition, and development. Lancet.

[17] Wang D, Stewart D (2013). The implementation and effectiveness of school-based nutrition promotion programmes using a health-promoting schools approach: a systematic review. Public Health Nutr.

[18] Kyere P, Veerman JL, Lee P, Stewart DE (2020). Effectiveness of school-based nutrition interventions in sub-Saharan Africa: a systematic review. Public Health Nutr.

[19] Bhutta ZA, Lassi ZS, Bergeron G (2017). Delivering an action agenda for nutrition interventions addressing adolescent girls and young women: priorities for implementation and research. Ann NY Acad Sci.

[20] Salam RA, Das JK, Irfan O, Ahmed W, Sheikh SS, Bhutta ZA (2020). Effects of preventive nutrition interventions among adolescents on health and nutritional status in low- and middle-income countries: a systematic review. Campbell Syst Rev.

[21] Tamiru D, Argaw A, Gerbaba M, Nigussie A, Ayana G, Belachew T (2016). Improving dietary diversity of school adolescents through school based nutrition education and home gardening in Jimma Zone: Quasi-experimental design. Eat Behav.

[22] Dansa R, Reta F, Mulualem D, Henry CJ, Whiting SJ (2019). A nutrition education intervention to increase consumption of pulses showed improved nutritional status of adolescent girls in Halaba special district, Southern Ethiopia. Ecol Food Nutr.

[23] Food and Agricultural Organization of the UN (2016). FHI 360. Minimum dietary diversity for women: a guide for measurement. https://www.fao.org/nutrition/assessment/tools/minimum-dietary-diversity-women/en/.

[24] Global Diet Quality Project (2022). DQQ Tools. https://www.globaldietquality.org/dqq.

[25] Demographic and Health Surveys Wealth Index Construction. https://dhsprogram.com/topics/wealth-index/Wealth-Index-Construction.cfm.

[26] Coates J, Swindale A, Bilinsky P (August, 2007). Household Food Insecurity Access Scale (HFIAS) for measurement of food access: indicator guide (v.3). https://www.fantaproject.org/sites/default/files/resources/HFIAS_ENG_v3_Aug07.pdf.

[27] Halala Handiso Y, Belachew T, Abuye C, Workicho A (2020). Low dietary diversity and its determinants among adolescent girls in Southern Ethiopia. Cogent Food Agric.

[28] Kim SS, Nguyen PH, Tran LM, Alayon S, Menon P, Frongillo EA (2019). Different combinations of behavior change interventions and frequencies of interpersonal contacts are associated with infant and young child feeding practices in Bangladesh, Ethiopia, and Vietnam. Curr Dev Nutr.

[29] Lamstein S, Stillman T, Koniz-Booher P (August, 2014). Evidence of effective approaches to social and behavior change communication for preventing and reducing stunting and anemia: report from a systematic literature review. https://www.spring-nutrition.org/sites/default/files/publications/series/spring_sbcc_lit_review.pdf.

