# Supplementary material for: Feasibility and impact of school-based nutrition education interventions on the diets of adolescent girls in Ethiopia: a non-masked, cluster-randomised, controlled trial
Source: Lancet Child Adolesc Health. 2023 Oct;7(10):686–96. doi: 10.1016/S2352-4642(23)00168-2 (PMC10509035; doi:10.1016/S2352-4642(23)00168-2)
Supplement: Supplementary appendix [file mmc1.pdf]

THE LANCET
Child & Adolescent Health

Supplementary appendix

This appendix formed part of the original submission and has been peer reviewed. We post it as supplied by the authors.

Supplement to: Kim SS, Sununtnasuk C, Berhane HY, et al. Feasibility and impact of school-based nutrition education interventions on the diets of adolescent girls in Ethiopia: a non-masked, cluster-randomised, controlled trial. *Lancet Child Adolesc Health* 2023; published online Sept 1. https://doi.org/10.1016/S2352-4642(23)00168-2.

SUPPLEMENTARY APPENDIX

Supplemental Table 1. CONSORT 2010 checklist with extensions for cluster randomized trials

Section/Topic	Item No	Standard Checklist item	Extension for cluster designs	Page No
**Title and abstract**				
	1a	Identification as a randomised trial in the title	Identification as a cluster randomised trial in the title	1 (Title)
	1b	Structured summary of trial design, methods, results, and conclusions (for specific guidance see CONSORT for abstracts)[i,ii]	See table 2	3 (Abstract)
**Introduction**				
Background and objectives	2a	Scientific background and explanation of rationale	Rationale for using a cluster design	8-9, 11-12 (whole school interventions described)
	2b	Specific objectives or hypotheses	Whether objectives pertain to the cluster level, the individual participant level or both	7
**Methods**				
Trial design	3a	Description of trial design (such as parallel, factorial) including allocation ratio	Definition of cluster and description of how the design features apply to the clusters	8-9
	3b	Important changes to methods after trial commencement (such as eligibility criteria), with reasons		None
Participants	4a	Eligibility criteria for participants	Eligibility criteria for clusters	9
	4b	Settings and locations where the data were collected		8
Interventions	5	The interventions for each group with sufficient details to allow replication, including how and when they were actually administered	Whether interventions pertain to the cluster level, the individual participant level or both	11-12, Supplemental Table 3
Outcomes	6a	Completely defined pre-specified primary and secondary outcome measures, including how and when they were assessed	Whether outcome measures pertain to the cluster level, the individual participant level or both	12-13
	6b	Any changes to trial outcomes after the trial commenced, with reasons		N/A
Sample size	7a	How sample size was determined	Method of calculation, number of clusters(s) (and whether equal or unequal cluster sizes are assumed), cluster size, a coefficient of intracluster	13-14

Section/Topic	Item No	Standard Checklist item	Extension for cluster designs	Page No
			correlation (ICC or *k*), and an indication of its uncertainty	
	7b	When applicable, explanation of any interim analyses and stopping guidelines		N/A
**Randomisation:**				
Sequence generation	8a	Method used to generate the random allocation sequence		10
	8b	Type of randomisation; details of any restriction (such as blocking and block size)	Details of stratification or matching if used	10
Allocation concealment mechanism	9	Mechanism used to implement the random allocation sequence (such as sequentially numbered containers), describing any steps taken to conceal the sequence until interventions were assigned	Specification that allocation was based on clusters rather than individuals and whether allocation concealment (if any) was at the cluster level, the individual participant level or both	10
Implementation	10	Who generated the random allocation sequence, who enrolled participants, and who assigned participants to interventions	Replace by 10a, 10b and 10c	(below)
	10a		Who generated the random allocation sequence, who enrolled clusters, and who assigned clusters to interventions	10
	10b		Mechanism by which individual participants were included in clusters for the purposes of the trial (such as complete enumeration, random sampling)	10-11
	10c		From whom consent was sought (representatives of the cluster, or individual cluster members, or both), and whether consent was sought before or after randomisation	9
Blinding	11a	If done, who was blinded after assignment to interventions (for example, participants, care providers, those assessing outcomes) and how		N/A
	11b	If relevant, description of the similarity of interventions		N/A
Statistical methods	12a	Statistical methods used to compare groups for primary and secondary outcomes	How clustering was taken into account	14-15
	12b	Methods for additional analyses, such as subgroup analyses and adjusted analyses		14-15
**Results**				

Section/Topic	Item No	Standard Checklist item	Extension for cluster designs	Page No
Participant flow (a diagram is strongly recommended)	13a	For each group, the numbers of participants who were randomly assigned, received intended treatment, and were analysed for the primary outcome	For each group, the numbers of clusters that were randomly assigned, received intended treatment, and were analysed for the primary outcome	Figure 1
	13b	For each group, losses and exclusions after randomisation, together with reasons	For each group, losses and exclusions for both clusters and individual cluster members	Figure 1
Recruitment	14a	Dates defining the periods of recruitment and follow-up		9
	14b	Why the trial ended or was stopped		N/A
Baseline data	15	A table showing baseline demographic and clinical characteristics for each group	Baseline characteristics for the individual and cluster levels as applicable for each group	Table 1
Numbers analysed	16	For each group, number of participants (denominator) included in each analysis and whether the analysis was by original assigned groups	For each group, number of clusters included in each analysis	Tables 1-3
Outcomes and estimation	17a	For each primary and secondary outcome, results for each group, and the estimated effect size and its precision (such as 95% confidence interval)	Results at the individual or cluster level as applicable and a coefficient of intracluster correlation (ICC or k) for each primary outcome	15-16
	17b	For binary outcomes, presentation of both absolute and relative effect sizes is recommended		15-16
Ancillary analyses	18	Results of any other analyses performed, including subgroup analyses and adjusted analyses, distinguishing pre-specified from exploratory		16-17
Harms	19	All important harms or unintended effects in each group (for specific guidance see CONSORT for harms[iii])		12
**Discussion**				
Limitations	20	Trial limitations, addressing sources of potential bias, imprecision, and, if relevant, multiplicity of analyses		20-21
Generalisability	21	Generalisability (external validity, applicability) of the trial findings	Generalisability to clusters and/or individual participants (as relevant)	20
Interpretation	22	Interpretation consistent with results, balancing benefits and harms, and considering other relevant evidence		17-20
Other information				

Section/Topic	Item No	Standard Checklist item	Extension for cluster designs	Page No
Registration	23	Registration number and name of trial registry		3, 8
Protocol	24	Where the full trial protocol can be accessed, if available		10
Funding	25	Sources of funding and other support (such as supply of drugs), role of funders		4, 10

**Extension of CONSORT for abstracts to report on cluster randomised trials**			
**Item**	**Standard Checklist item**	**Extension for cluster trials**	**Line number**
Title	Identification of study as randomised	Identification of study as cluster randomised	6
Trial design	Description of the trial design (e.g. parallel, cluster, non-inferiority)		6
**Methods**			
Participants	Eligibility criteria for participants and the settings where the data were collected	Eligibility criteria for clusters	6-8, 10-11
Interventions	Interventions intended for each group		11-14
Objective	Specific objective or hypothesis	Whether objective or hypothesis pertains to the cluster level, the individual participant level or both	4-5
Outcome	Clearly defined primary outcome for this report	Whether the primary outcome pertains to the cluster level, the individual participant level or both	15-16
Randomization	How participants were allocated to interventions	How clusters were allocated to interventions	7-8 (details provided in the manuscript)
Blinding (masking)	Whether or not participants, care givers, and those assessing the outcomes were blinded to group assignment		6
**Results**			
Numbers randomized	Number of participants randomized to each group	Number of clusters randomized to each group	22, 6-7
Recruitment	Trial status[1]		
Numbers analysed	Number of participants analysed in each group	Number of clusters analysed in each group	22, 6-7
Outcome	For the primary outcome, a result for each group and the estimated effect size and its precision	Results at the cluster or individual participant level as applicable for each primary outcome	25-27
Harms	Important adverse events or side effects		None
Conclusions	General interpretation of the results		28-31

[1] Relevant to Conference Abstracts

Trial registration	Registration number and name of trial register		20
Funding	Source of funding		32-33

REFERENCES

[i] Hopewell S, Clarke M, Moher D, Wager E, Middleton P, Altman DG, et al. CONSORT for reporting randomised trials in journal and conference abstracts. *Lancet* 2008, 371:281-283

[ii] Hopewell S, Clarke M, Moher D, Wager E, Middleton P, Altman DG at al (2008) CONSORT for reporting randomized controlled trials in journal and conference abstracts: explanation and elaboration. *PLoS Med* 5(1): e20

[iii] Ioannidis JP, Evans SJ, Gotzsche PC, O'Neill RT, Altman DG, Schulz K, Moher D. Better reporting of harms in randomized trials: an extension of the CONSORT statement. *Ann Intern Med* 2004; 141(10):781-788.

Supplemental Table 2. School food environment and exposure to food advertisements by study arm and survey round

At baseline, three schools (out of 54) provided any food at school and two schools had a canteen, only one of which offered any food on school premises. Immediately outside the schools, about 57 percent had at least one food point (vendor/stand, shop, or restaurant), which mostly sold candies, cookies, biscuits, or packaged snacks (77 percent) and soda or sugar-sweetened beverages (65 percent). At endline, only one school in the intervention area had a canteen but no food items were observed, and nearly all the schools (96 percent) had at least one food point with mostly sold sweets and soda/sugar-sweetened drinks.

Within the past month at baseline, 13 percent of adolescent girls reported seeing or hearing any food advertisement. At endline, about a quarter of adolescent girls had seen or heard a food advertisement in the past 3 months.

Results are presented below:

School indicator	Baseline		Endline	
	Intervention	Control	Intervention	Control
	N=27	N=27	N=27	N=27
**Free meals/food provided at school**	3 (11%)	0 (0%)	0 (0%)	0 (0%)
**School has a canteen**	1 (4%)	1 (4%)	1 (4%)	0 (0%)
**1+ food points within a 1-minute walk of the school**	17 (63%)	14 (52%)	26 (96%)	26 (96%)
**Number of food points within a 1-minute walk**	1.9±1.3	1.7±1.1	2.0+1.5	1.4+0.7
**Food point sells**				
Hot cooked food	5/17 (29%)	1/14 (7%)	---	---
Fruits or vegetables	1/17 (6%)	0/14 (0%)	4/26 (15%)*	0/26 (0%)
Candy, cookies, biscuits, or packaged snacks	12/17 (71%)	12/14 (86%)	---	---
Soda or sugar-sweetened drinks	10/17 (59%)	10/14 (71%)	15/26 (58%)	13/26 (50%)
Unsweetened drinks	2/17 (12%)	2/14 (14%)	2/26 (8%)	1/26 (4%)
Fried foods[1]	---	---	7/26 (27%)	7/26 (27%)
Packaged salty snacks[2]	---	---	4/26 (15%)	7/26 (27%)
Sweets[2]	---	---	12/26 (46%)	17/26 (65%)
**Adolescent girl indicator**	**Baseline**		**Endline**	
	**Intervention**	**Control**	**Intervention**	**Control**
	**N=81**	**N=81**	**N=270**	**N=266**
**Exposure to food advertisements[3]**	14 (17%)	7 (9%)	94 (35%)***	36 (14%)

Values are mean ± SD or n (%). Differences between study arms at endline, accounting for clustering: *p<0.05, ***p < 0.001.
[1]Renamed variable at endline, replacing "hot cooked food" at baseline.
[2]Recategorized variables at endline, replacing "candy, cookies, biscuits, or packaged snacks" at baseline.
[3]Within the past month at baseline and within the past 3 months at endline.

Supplemental Table 3. Description of A&T interventions

Intervention	Description
**Core school-based interventions:**	
Classroom education about healthy diet and nutrition	Science teachers provide weekly classroom lessons related to dietary diversity, meal frequency, healthy food choices, and hand washing
Flag events or assemblies about healthy diet and nutrition	Weekly flag events or school assemblies led by principals to provide messages on dietary diversity, meal frequency, healthy food choices, and hand washing
School club sessions	Student club leaders and teacher club leaders promote adolescent nutrition during school club sessions (including nutrition clubs and girls' clubs).
Peer group mentoring on healthy diets and nutrition	Selected adolescent girls are mentored by science teachers as peer mentors and hold weekly group discussions with other girls to discuss nutrition, dietary diversity, meal frequency, healthy food choices and handwashing, and encourage discussions about nutrition with parents
BMI measurement and counseling	Trained science teachers take anthropometric measurements of adolescent girls to calculate their body mass index (BMI) and provide nutrition counseling at least once per semester
Parent-teacher meetings	Monthly parent-teacher meetings to inform and encourage parents about adolescent nutrition, dietary diversity, healthy snacks, and handwashing
**Secondary interventions:**	
Home visits by health workers	Home visits by HEWs and/or community volunteers to discuss adolescent nutrition, dietary diversity, meal frequency, healthy food choices, and handwashing with parents.
Community gatherings to discuss adolescent nutrition	Community gatherings by HEWs and meetings with religious and kebele leaders to discuss adolescent nutrition, dietary diversity, meal frequency, healthy food choices, and handwashing with parents.
**Capacity-building of service providers:**	
Training for school staff and other actors	Workshop on the adolescent nutrition interventions above for school principals, science teachers, HEWs, supervisors, and woreda officers
Supportive supervision for school staff and other actors	Biweekly supportive supervision on adolescent nutrition activities for schools and HEWs by school supervisors or woreda health/education office

BMI: body mass index; HEW: health extension worker

Supplemental Table 4. Association between reported exposure to number of intervention components and study outcomes in the intervention arm

		Minimum dietary diversity		High meal frequency (>3 times)		Junk food consumption	
	Intervention arm (N=270)	Adjusted[1] β /OR (95% CI)	p-value	Adjusted[1] β /OR (95% CI)	p-value	Adjusted[1] β /OR (95% CI)	p-value
**Exposure to interventions (0-6)**							
0	14 (5%)	*Ref*		*Ref*		*Ref*	
1	6 (2%)	56.12 (11.15-282.62)	<0.001	2.18 (-0.15-4.51)	0.066	1.73 (0.28-10.86)	0.56
2	25 (9%)	94.00 (17.87-494.50)	<0.001	2.31 (0.03-4.58)	0.047	1.43 (0.30-6.70)	0.65
3	55 (20%)	63.49 (13.30-302.99)	<0.001	2.08 (0.16-4.00)	0.034	1.55 (0.44-5.41)	0.50
4	73 (27%)	60.98 (16.87-220.45)	<0.001	2.78 (0.65-4.91)	0.010	1.22 (0.48-3.08)	0.67
5	56 (21%)	52.08 (15.54-174.55)	<0.001	2.45 (0.20-4.69)	0.033	2.41 (0.96-6.07)	0.061
6	41 (15%)	49.43 (12.35-197.88)	<0.001	2.54 (0.30-4.78)	0.026	1.24 (0.42-3.66)	0.69

Differences between study arms in intent-to-treat analyses, accounting for clustering.
[1] Logistic regression, adjusted for adolescent age, region, household food security and wealth, clustered by school.

Supplemental Table 5. Association between reported exposure to types of intervention components and study outcomes in the intervention arm

	Intervention arm (N=270)	Minimum dietary diversity		High meal frequency (>3 times)		Junk food consumption	
		Adjusted[1] β /OR (95% CI)	p-value	Adjusted[1] β /OR (95% CI)	p-value	Adjusted[1] β /OR (95% CI)	p-value
**Exposure to interventions**							
Parents' meetings	145 (54%)	1.20 (0.61-2.36)	0.60	0.34 (-0.45-1.13)	0.40	0.88 (0.50-1.56)	0.67
Flag ceremonies	230 (85%)	5.38 (1.90-15.24)	0.002	1.30 (0.33-2.27)	0.009	0.90 (0.44-1.85))	0.78
Classroom lessons	226 (84%)	1.91 (0.59-6.21)	0.28	0.92 (-0.19-2.04)	0.11	1.12 (0.54-2.34)	0.76
Girls club	121 (45%)	0.97 (0.53-1.78)	0.92	0.31 (-0.23-0.86)	0.26	1.40 (0.78-2.52)	0.26
Peer mentorship	120 (44%)	0.89 (0.50-1.58)	0.70	0.36 (-0.16-0.88)	0.17	1.07 (0.59-1.95)	0.83
BMI measurements	197 (73%)	2.37 (1.16-4.85)	0.018	0.44 (-0.30-1.18)	0.24	1.21 (0.72-2.03)	0.47

Differences between study arms in intent-to-treat analyses, accounting for clustering: *p < 0.05, **p < 0.01, ***p < 0.001.
[1] Logistic regression, controlled for adolescent age, region, household food security and wealth, clustered by school.
